# Supplementary figures and images for: Self-standing zeolite foam monoliths with hierarchical micro–meso–macroporous structures
Source: R Soc Open Sci. 2020 Aug 12;7(8):200981. doi: 10.1098/rsos.200981 (PMC7481723; doi:10.1098/rsos.200981)

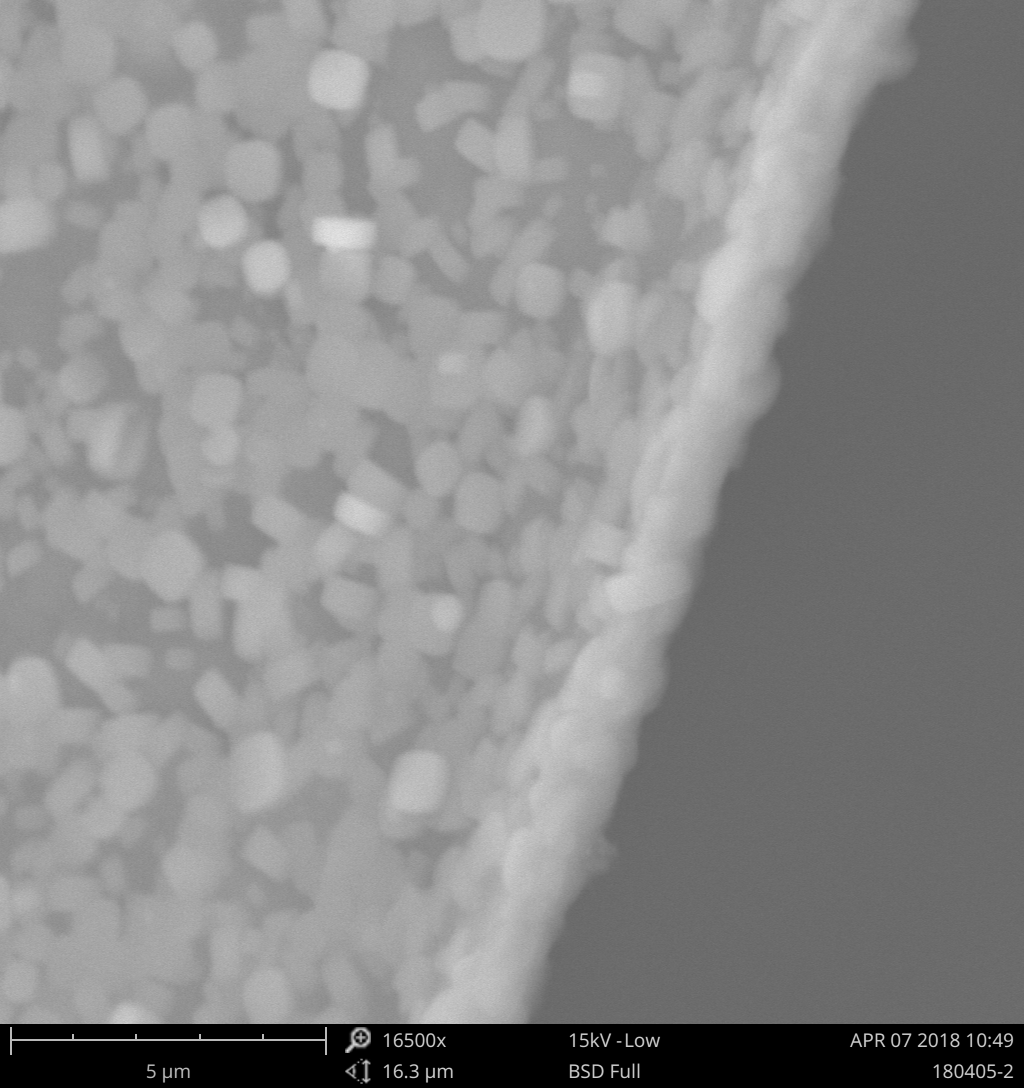

Supplement: Electronic Supplementary Material [file rsos200981supp1.zip › 180405-20018.tiff]

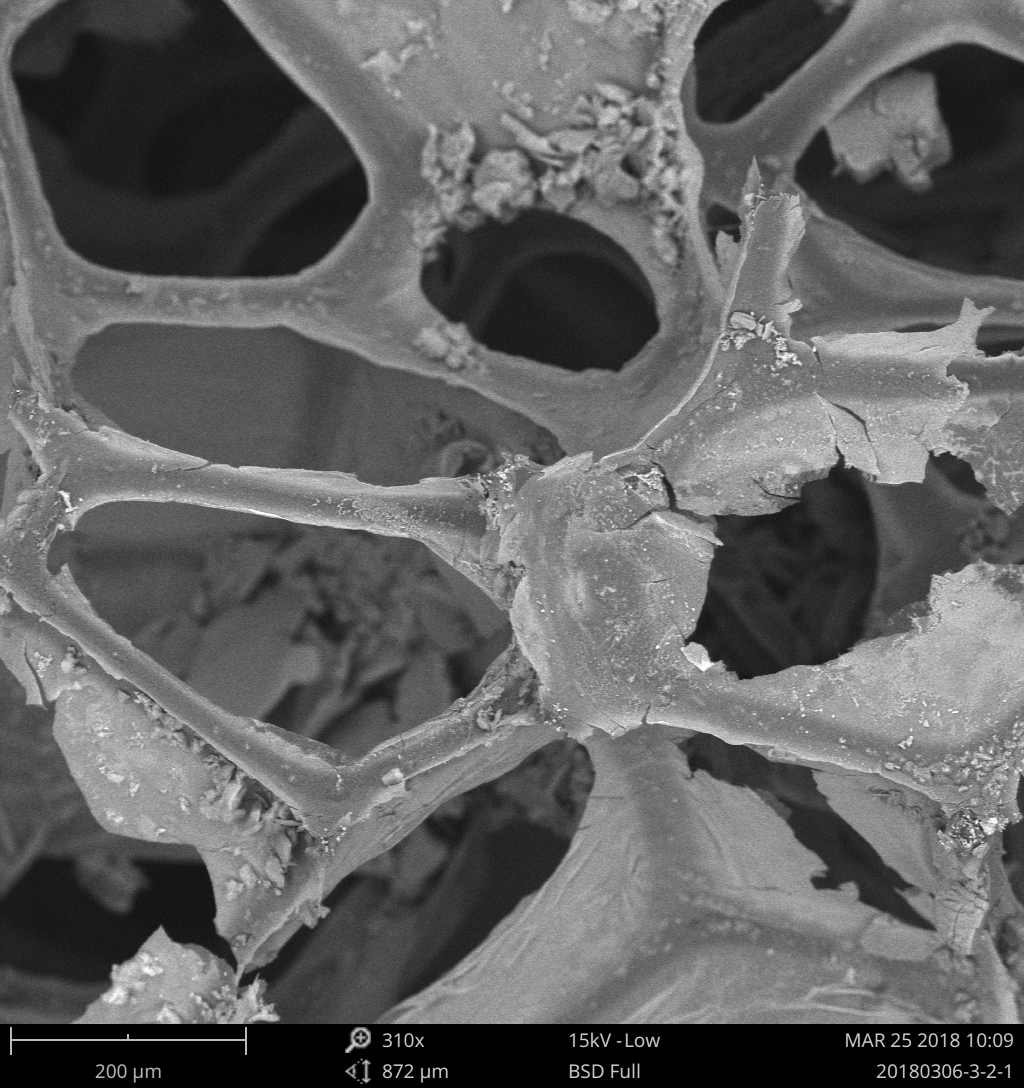

Supplement: Electronic Supplementary Material [file rsos200981supp1.zip › 20180306-3-2-10011.tiff]

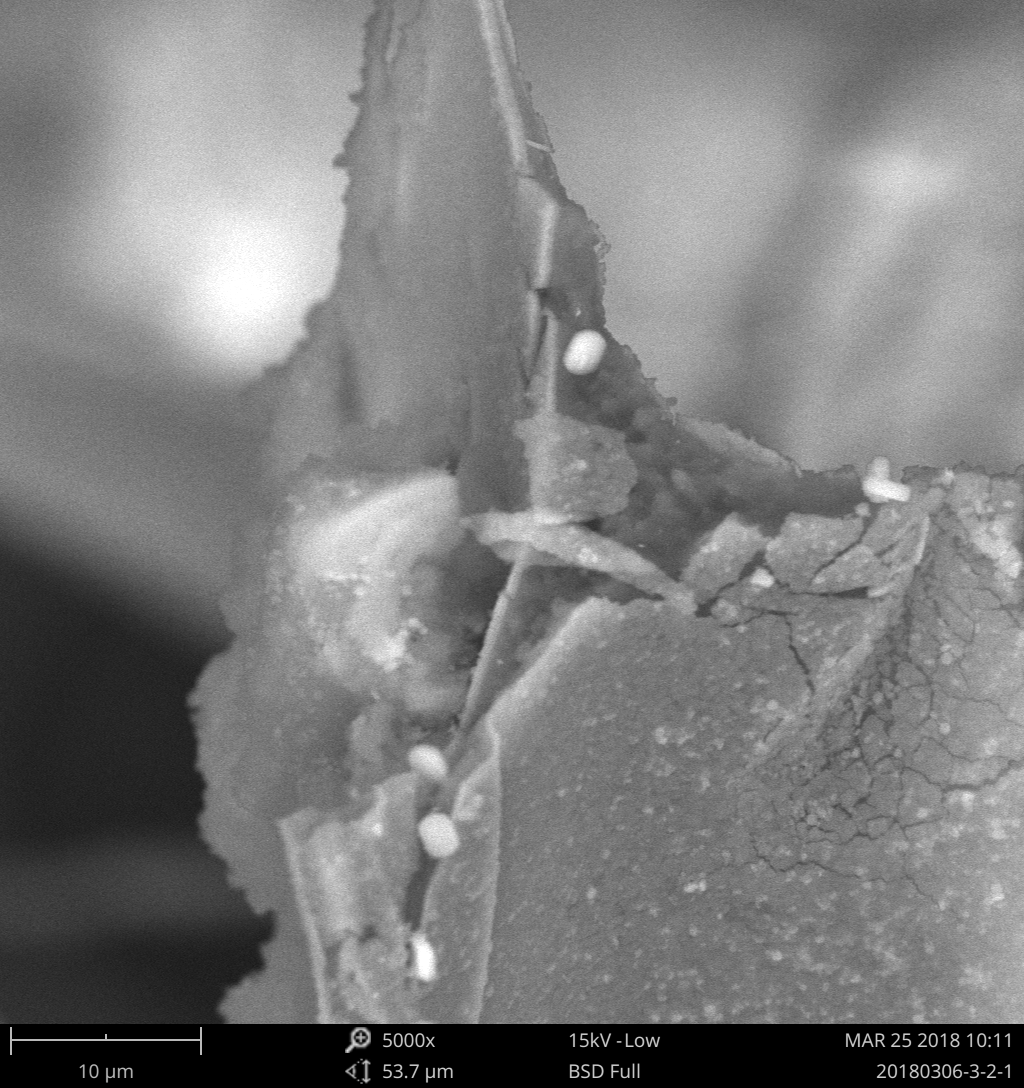

Supplement: Electronic Supplementary Material [file rsos200981supp1.zip › 20180306-3-2-10012.tiff]

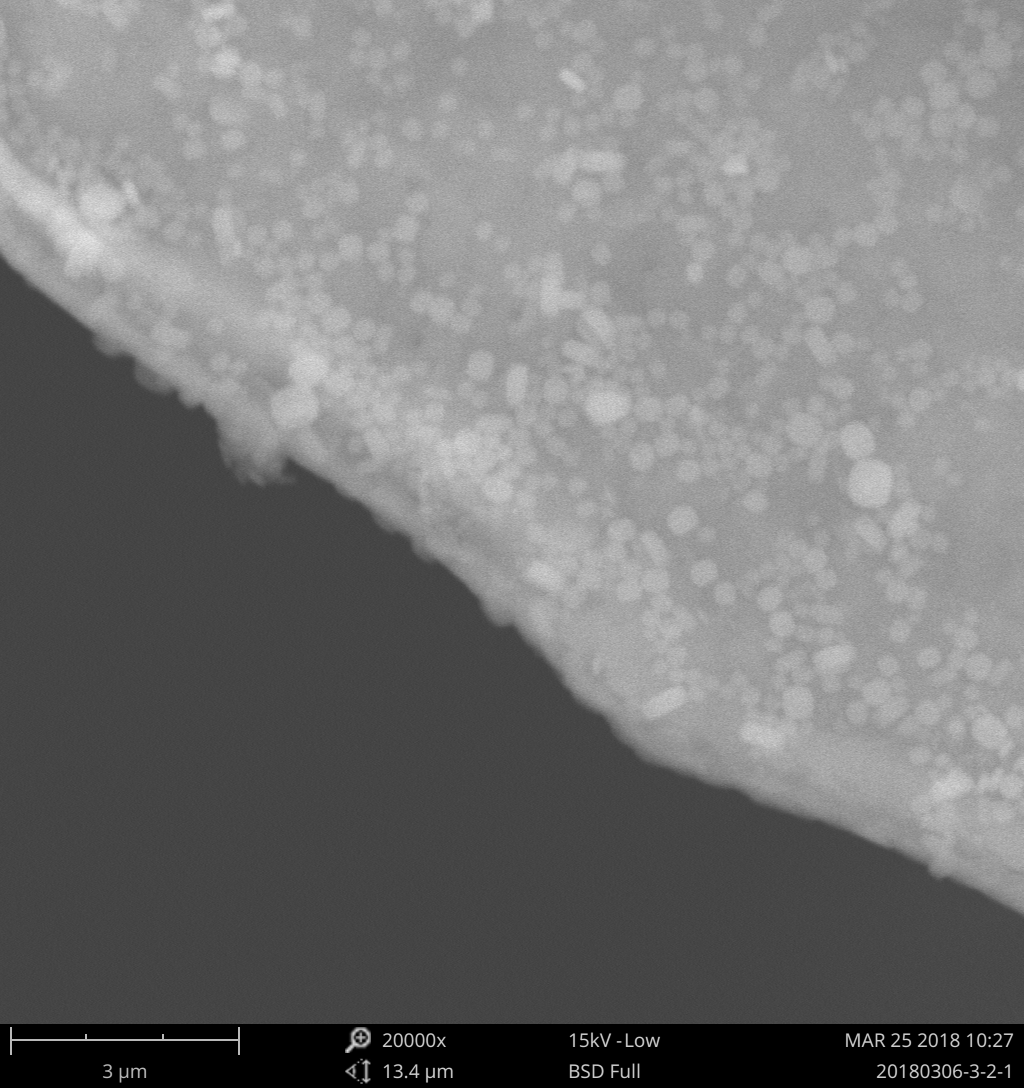

Supplement: Electronic Supplementary Material [file rsos200981supp1.zip › 20180306-3-2-10018.tiff]

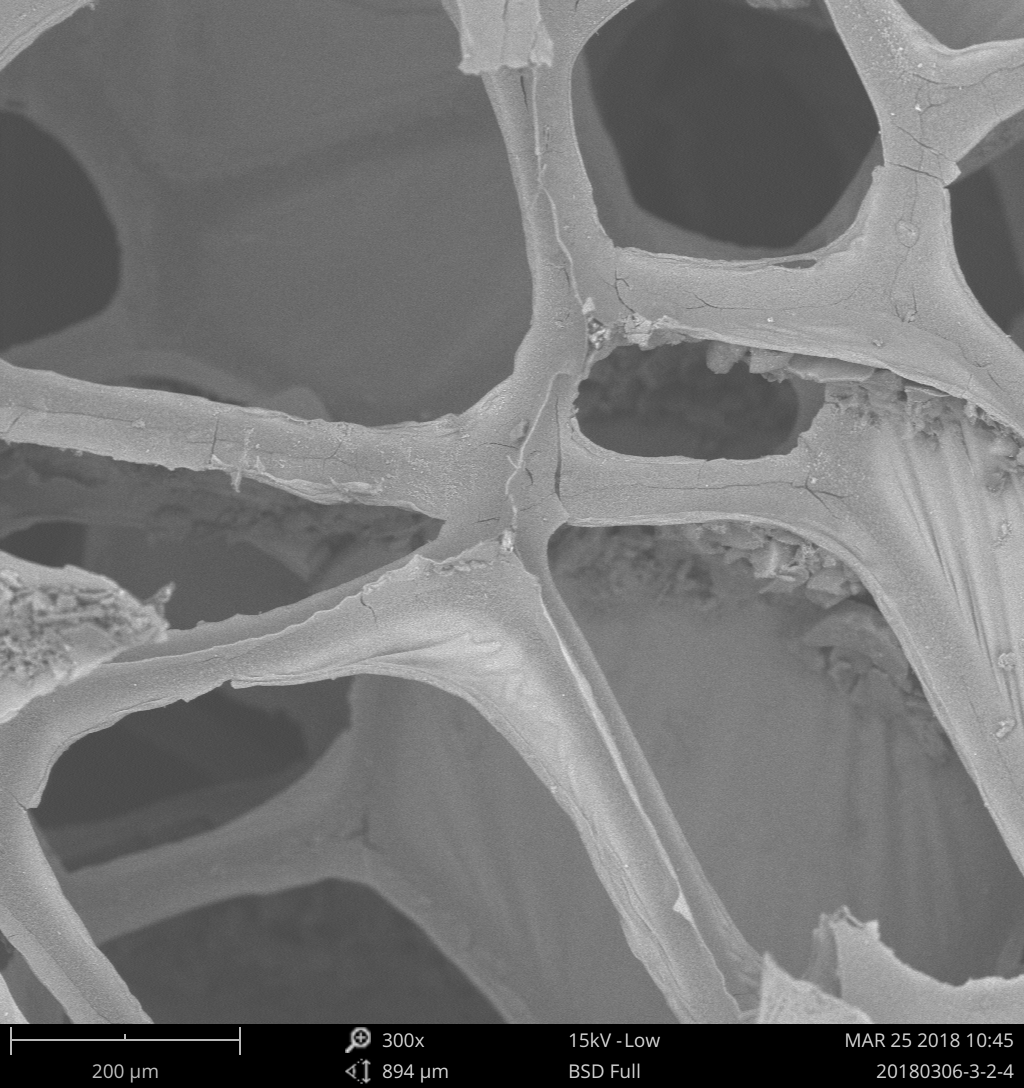

Supplement: Electronic Supplementary Material [file rsos200981supp1.zip › 20180306-3-2-40001--36h.tiff]

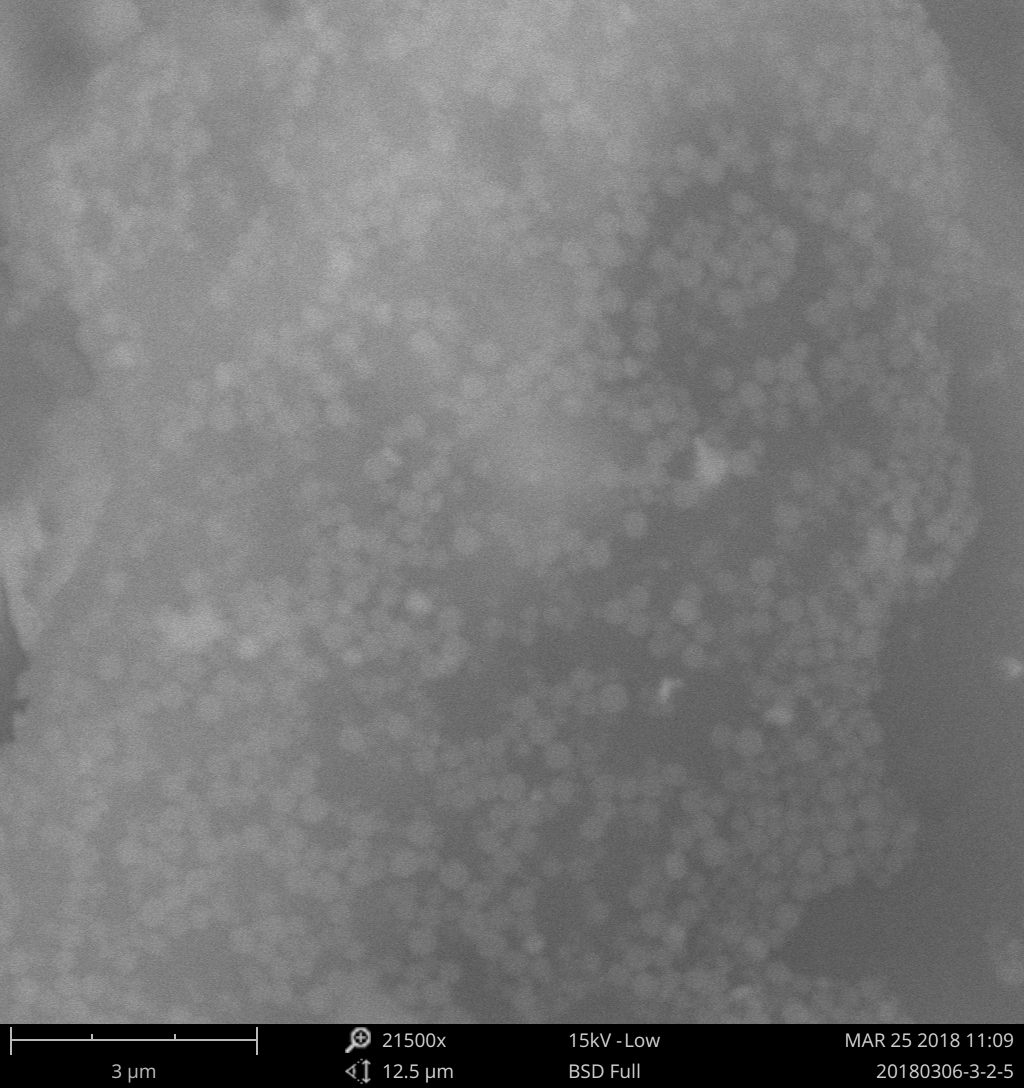

Supplement: Electronic Supplementary Material [file rsos200981supp1.zip › 20180306-3-2-50014.tiff]

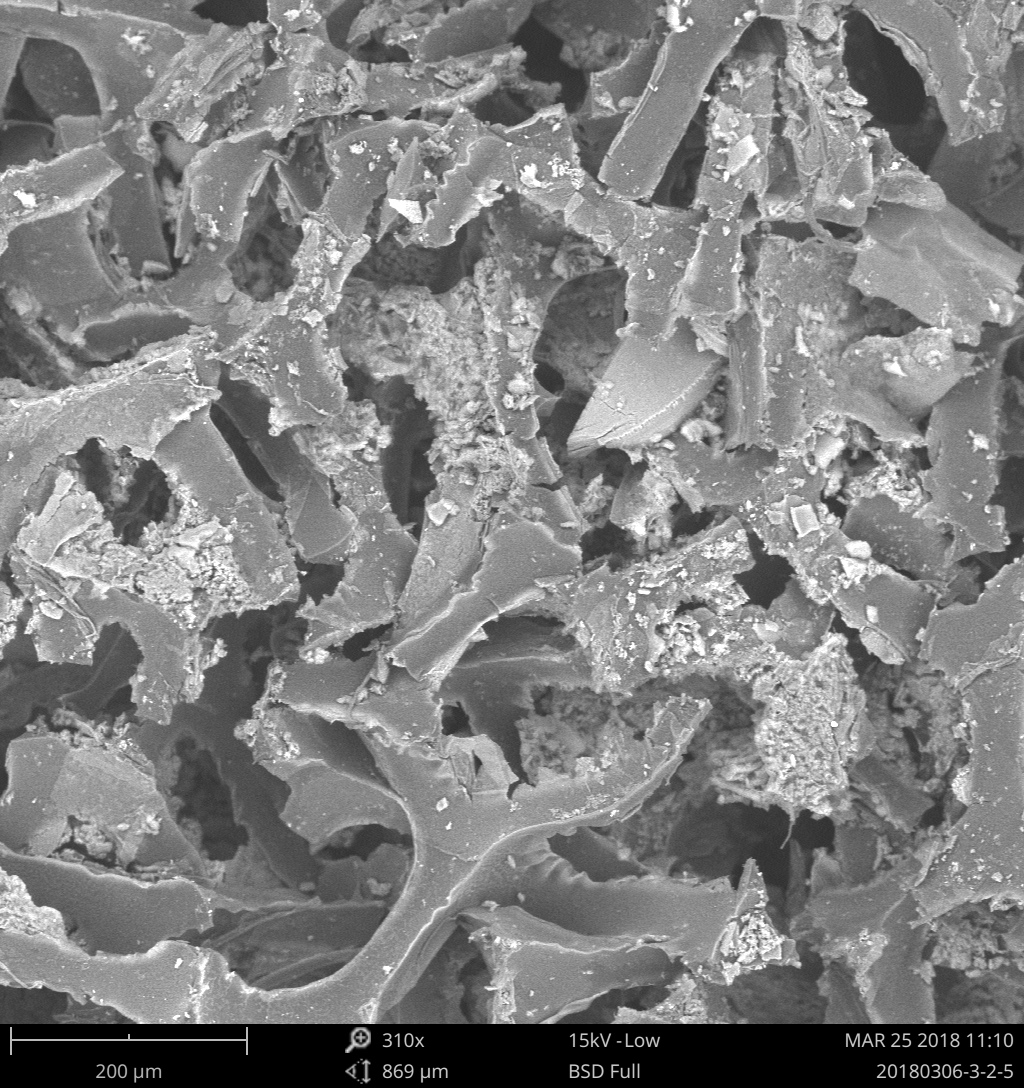

Supplement: Electronic Supplementary Material [file rsos200981supp1.zip › 20180306-3-2-50015.tiff]

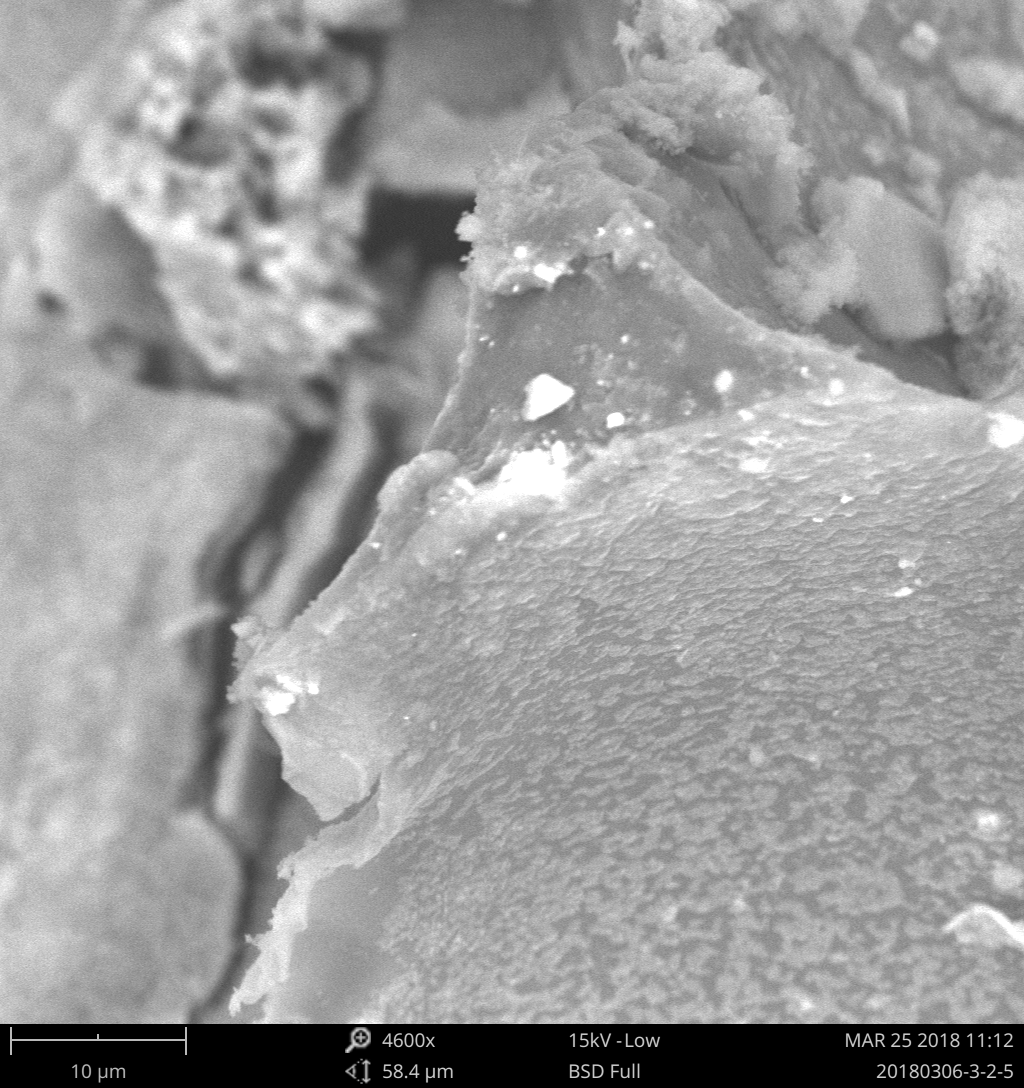

Supplement: Electronic Supplementary Material [file rsos200981supp1.zip › 20180306-3-2-50019.tiff]

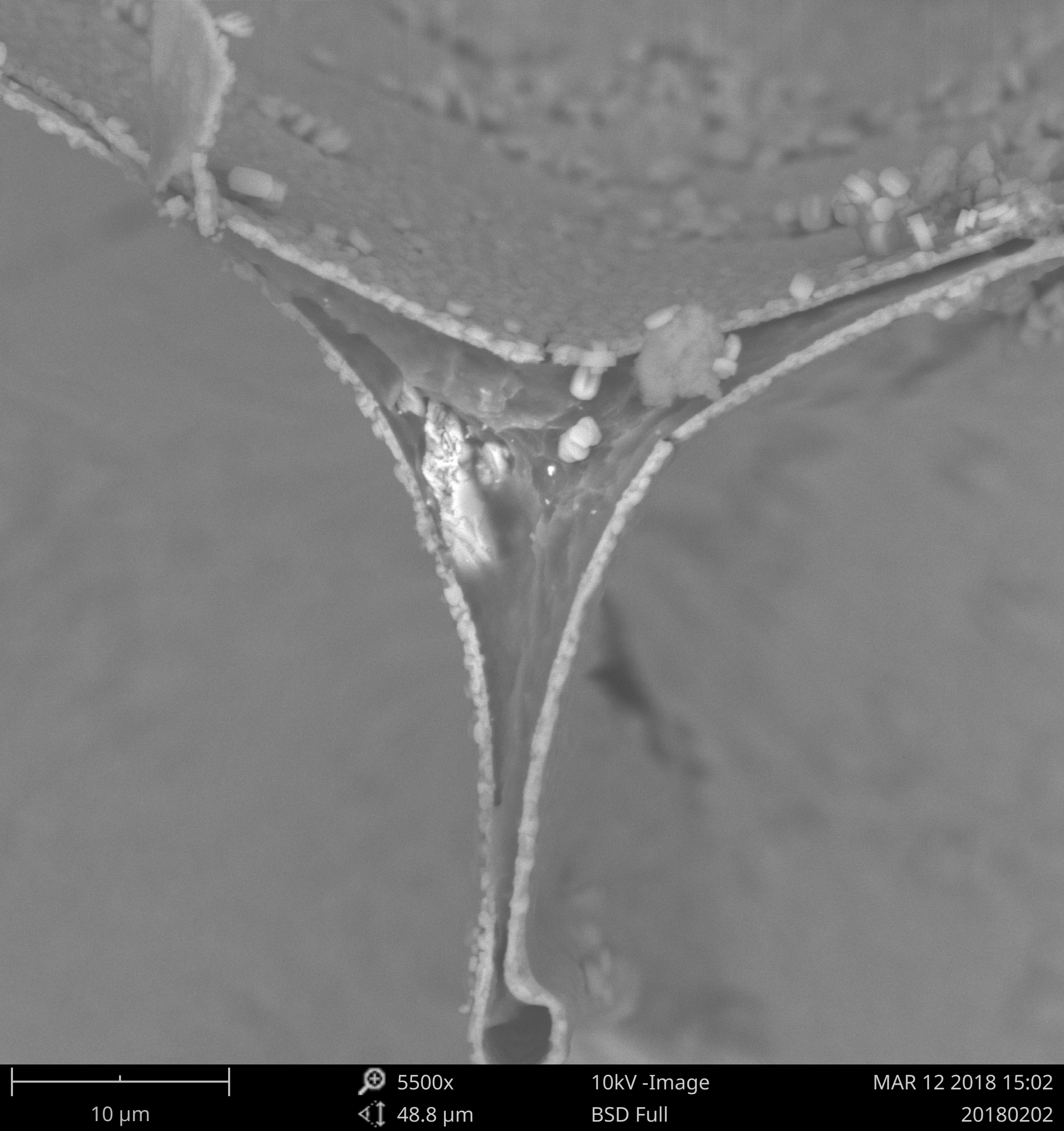

Supplement: Electronic Supplementary Material [file rsos200981supp1.zip › 201802020016.tiff]

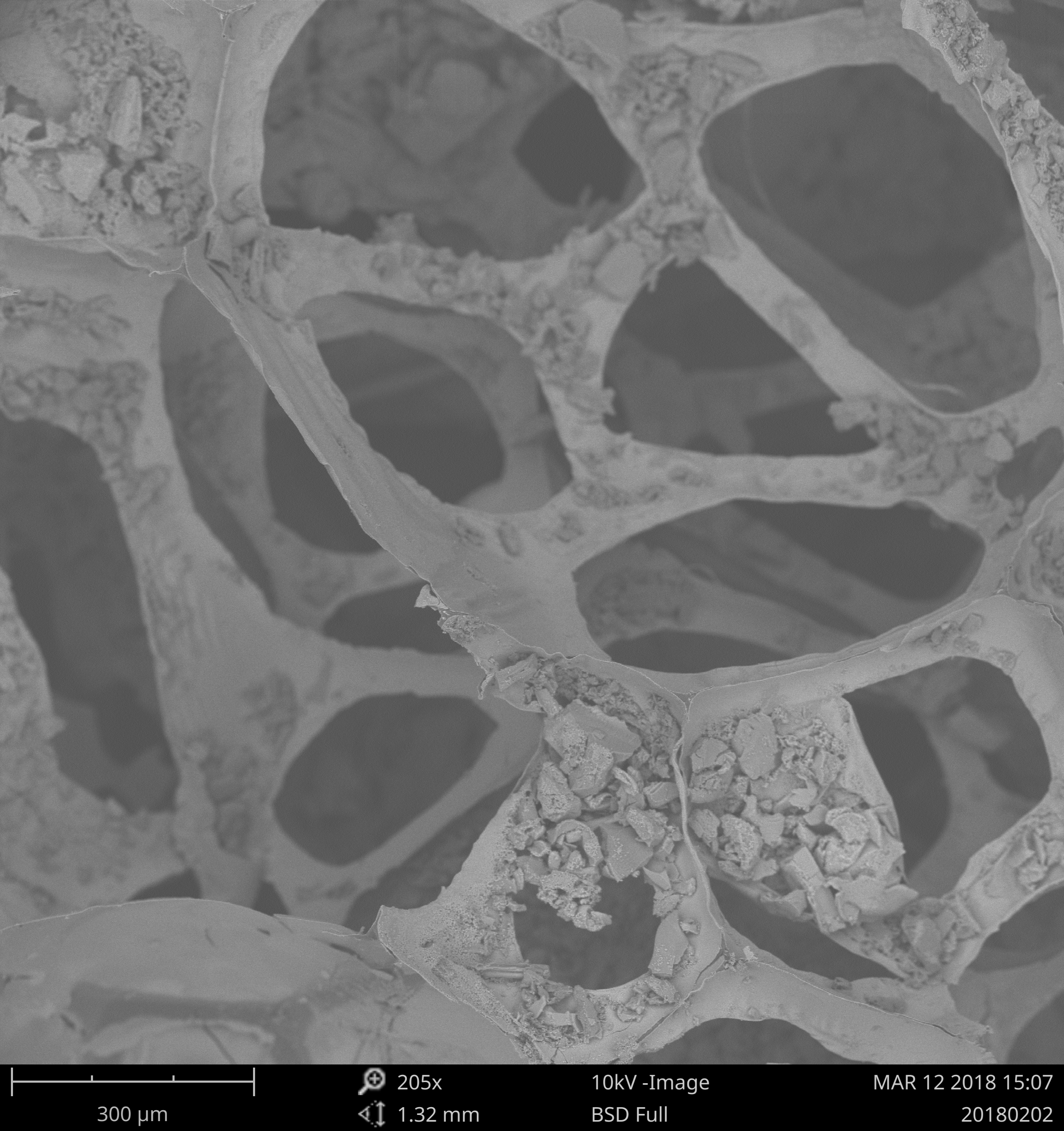

Supplement: Electronic Supplementary Material [file rsos200981supp1.zip › 201802020022.tiff]
